# Chronic Intermittent Hypoxia Induces the Long-Term Facilitation of Genioglossus Corticomotor Activity

**DOI:** 10.1155/2018/5941429

**Published:** 2018-04-23

**Authors:** Ying Zou, Wei Wang, Xinshi Nie, Jian Kang

**Affiliations:** Institute of Respiratory Disease, The First Hospital of China Medical University, Shenyang, China

## Abstract

Obstructive sleep apnea (OSA) is characterized by the repetitive collapse of the upper airway and chronic intermittent hypoxia (CIH) during sleep. It has been reported that CIH can increase the EMG activity of genioglossus in rats, which may be related to the neuromuscular compensation of OSA patients. This study aimed to explore whether CIH could induce the long-term facilitation (LTF) of genioglossus corticomotor activity. 16 rats were divided into the air group (*n*=8) and the CIH group (*n*=8). The CIH group was exposed to hypoxia for 4 weeks; the air group was subjected to air under identical experimental conditions in parallel. Transcranial magnetic stimulation (TMS) was applied every ten minutes and lasted for 1 h/day on the 1st, 3rd, 7th, 14th, 21st, and 28th days of air/CIH exposure. Genioglossus EMG was also recorded at the same time. Compared with the air group, the CIH group showed decreased TMS latency from 10 to 60 minutes on the 7th, 14th, 21st, and 28th days. The increased TMS amplitude lasting for 60 minutes was only observed on the 21st day. Genioglossus EMG activity increased only on the 28th day of CIH. We concluded that CIH could induce LTF of genioglossus corticomotor activity in rats.

## 1. Introduction

Obstructive sleep apnea syndrome (OSAS) is mainly manifested as the recurrent collapse of the upper airway during sleep. Considering that the intermittent hypoxia (IH) is a hallmark of sleep apnea, the IH-induced long-term facilitation (LTF) was an intriguing finding [1]. As the most common model of respiratory plasticity, LTF is characterized by an escalation of the respiratory motor activation during the normoxia period following the intermittent hypoxia and by a long-lasting enhancement in respiratory activity for 90 minutes after repetitive IH exposure [2]. LTF, as a kind of plasticity of respiratory motoneuron activity, has many forms including phrenic nerve activity LTF (pLTF), hypoglossal nerve activity LTF (hLTF), ventilation LTF (vLTF), and sensory LTF (sLTF) [3]. It was postulated that the exposure to IH throughout the night might elicit the LTF of respiratory and upper airway muscle activities, which could mitigate the cyclical events characterized by breathing instability. Indeed, Mckay et al. observed that episodic hypoxia evoked the LTF of genioglossus EMG in newborn rats, indicating that genioglossus LTF played an important role in preventing the upper airway from collapse [4]. Mateika et al. reported that the exposure to mild IH resulted in a reduction in the therapeutic continuous positive airway pressure that required eliminating breathing events [5]. Thus, it is reasonable to assume that upper airway muscle LTF might be a protective mechanism against apnea. This has also been confirmed by Powell and Mitchell, who observed that ventilatory LTF could maintain respiratory stability especially during sleep [6]. Nevertheless, the occurrence of LTF at the level of the genioglossus corticomotor area remains unknown, especially during and after the daily exposure to CIH. It could be assumed that this IH-induced plasticity might present as a dynamic alteration of the corticomotor excitability and the EMG activity of genioglossus over time.

Transcranial magnetic stimulation (TMS) technique can be used to explore the cortical spinal cord conduction pathway of the skeletal muscle. With the technique of transcranial magnetic stimulation (TMS), Wang et al. studied the TMS response of the genioglossus corticomotor area in normal and OSAS patients [7, 8]. Sériès et al. further studied genioglossus corticomotor activity in awake OSAS patients and observed negative correlation between the apnea-hypopnea index (AHI) and TMS latency [8]. Our previous study also found an increased activity of the genioglossus corticomotor area in the rats during 4 weeks of CIH [9]. These above findings suggested that OSAS patients had central compensation during wakefulness, which might be related to the exposure to CIH. In this regard, this study aimed to explore the alteration of genioglossus corticomotor excitability using TMS and its EMG activity over time after IH stimulation at different stages of CIH in rats.

## 2. Animals and Materials

### 2.1. Animals

Sixteen adult male Wistar rats (280–300 g, 8 weeks old) were provided by Liaoning Changsheng Biotechnology Company (Benxi City, China). The rats were randomly divided into two groups: the CIH group (*n*=8) and the air group (*n*=8). All animals were housed in polypropylene cages with a capacity of 15 cm × 20 cm × 20 cm, were given free access to water and food, and were housed under controlled conditions (temperature 24 ± 2°C and relative air humidity 40%) with a 12 : 12 h light-dark cycle (lights on at 8:00 am and lights off at 8:00 pm). All procedures were performed in accordance with the National Institutes of Health Guide for Care and Use of Laboratory Animals and were approved by the Animal Ethics and Use Committee of China Medical University.

### 2.2. Chronic Intermittent Hypoxia

The rats in the CIH group were subjected to oxycycler (OxyCycler model A84XOV; BioSpherix, NY) hypoxia (10% O_2_ in N_2_ for 45 s) and normoxia (21% O_2_ in N_2_ for 72 s) every 180 s for 8 h/d (from 8 am to 4 pm), for 4 consecutive weeks. The rats in the air group were subjected to air under identical experimental conditions in parallel. The O_2_ concentration was continuously measured by an O_2_ analyzer and was changed by a computerized system controlling the gas outlets (shown as the Supplementary Figure 2). On the 1st, 3rd, 7th, 14th, 21st, and 28th days of the experiments in all groups, the last 30 min could be considered as acute intermittent hypoxia stimulation, as previously mentioned [10].

### 2.3. Transcranial Magnetic Stimulation (TMS)

Transcranial magnetic stimulation was carried out on the 1st, 3rd, 7th, 14th, 21st, and 28th days of the experiments in all groups. The rats were supinely positioned on a wooden board, and their heads, bodies, and limbs were restrained. Then, single-pulse TMS was performed by a Magstim 200 stimulator (Magstim, Whiteland, Dyfed, UK) with a 25 mm figure-eight coil. The coil was held against the rat's head. The TMS response of the genioglossus corticomotor area was described in terms of the corresponding motor-evoked potential (MEP) amplitude and latency. The optimal coil position was defined as that with the best response to TMS (the highest MEP amplitude and the shortest MEP latency). Briefly, the optimal stimulation site was 3.0–5.0 mm rostral to bregma and 2.0–4.0 mm lateral from the midline (shown as the supplementary Figure 1). The site was clearly marked with indelible ink, and the position of the coil was kept constant at the stimulation site by using a high-precision multipositional support consisting of two articulated arms. For each position, three stimuli were applied with at least 30 s interval and averaged for the mean MEP response. All TMSs were delivered at the end of normal expiration in the rats after intermittent hypoxia. The respiratory phase was determined by the detection of abdominal movement [9]. A concentric needle electrode (NM-131 T, Nihon Kohden, Japan) was inserted into the genioglossus. MEP was recorded using a computer software package (AxoScope software 9.0, Axon Instruments, Inc., USA). MEP latency was defined as the time up to the first deflection from baseline following TMS, and its amplitude was measured from the peak-to-peak TMS response. The latency and amplitude of the TMS response were recorded at 10 min, 20 min, 30 min, 40 min, 50 min, and 60 min after intermittent hypoxia, respectively, on each day (Supplementary Tables 1–3). The rats were appropriately anesthetized with sodium pentobarbital (40 mg/kg). The methodology is described in detail in our previous studies [9, 11].

### 2.4. Genioglossus EMG

The EMG activity of genioglossus was recorded by inserting a concentric needle electrode (NM-131T, Nihon Kohden, Japan) into the genioglossus. It was filtered (300–10,000 Hz), rectified, and integrated (Paynter Filter, BAK Electronics, Mount Airy, MD; time constant 200 ms). The integrated signals were digitized and acquired with computer software (LabVIEW 8.0, National Instruments) and analyzed with a customized program [3, 10]. This software determined the amplitude and timing of integrated EMG activity during early inspiration, from which the EMG of genioglossus was calculated. Genioglossus EMG was recorded at 10 min, 20 min, 30 min, 40 min, 50 min, and 60 min after the exposure to intermittent hypoxia, respectively, on each day.

Cardiorespiratory parameters (heart rate and central respiratory rate) were calculated over 10 min interval prior to the performance of TMS. If needed, supplemental doses of sodium pentobarbital were administered as increments until the absence of a toe-pitch withdrawal reflex.

### 2.5. Statistics Analysis

The results were reported as means ± SD. For the analysis of genioglossus EMG activities and TMS responses among different groups at the same time point, a repeated measure two-way ANOVA followed by Dunnett's studentized test was applied with two factors: times and treatments. All data were analyzed using SPSS 17.0 software. *p* < 0.05 was considered statistically significant.

## 3. Results

Cardiorespiratory parameters (heart rate and central respiratory rate) had no significance difference from 10 min to 60 min during the experiment.

### 3.1. EMG

**Figure 1:**
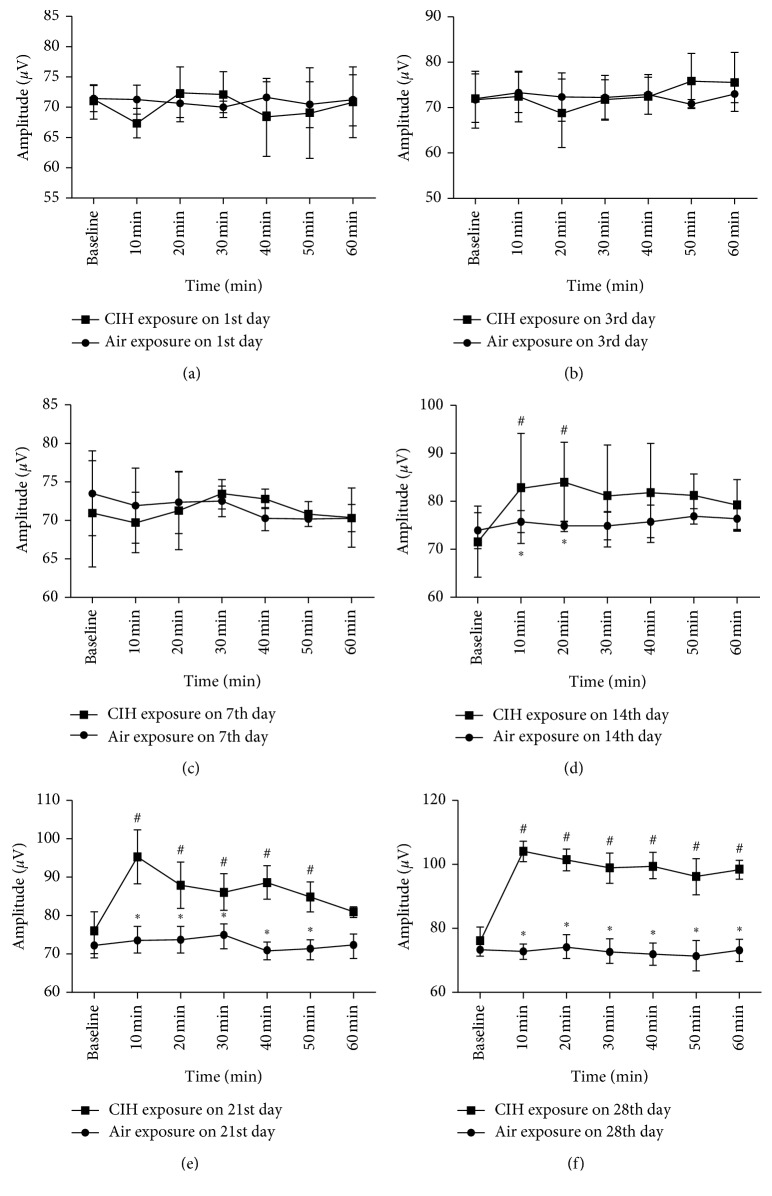
The comparison of genioglossus EMG activity at different time points after IH stimulation among different groups. (a–f) Values obtained on the 1st, 3rd, 7th, 14th, 21st, and 28th days of the daily air/CIH exposure. ^∗^Difference between AIR and CIH groups at the same time point. ^#^Difference between values detected at different time points in the CIH group.

Compared with the baseline value, the CIH group begun to show an increased EMG activity of the genioglossus at 10 and 20 min after hypoxia stimulation on the 14th day (71.63 ± 7.41 versus 82.62 ± 11.48 and 84 ± 8.29 for values at baseline, 10 min, and 20 min after IH, resp.; *p* < 0.05). Compared with the air group, the CIH group revealed the increased EMG activity of the genioglossus at 10 min and 20 min after hypoxia stimulation on the 14th day (75.78 ± 2.34 versus 82.62 ± 11.48 and 74.77 ± 1.08 versus 84 ± 8.29 for values at 10 min and 20 min after IH for the air and CIH groups, resp.; *p* < 0.05). This increment of EMG activity persisted within 30 min after the IH, as observed by a decline of the value at 30 min. At the 28th day, the increased genioglossus EMG activity lasted for 60 min (Figure 1).

### 3.2. TMS Responses

**Figure 2:**
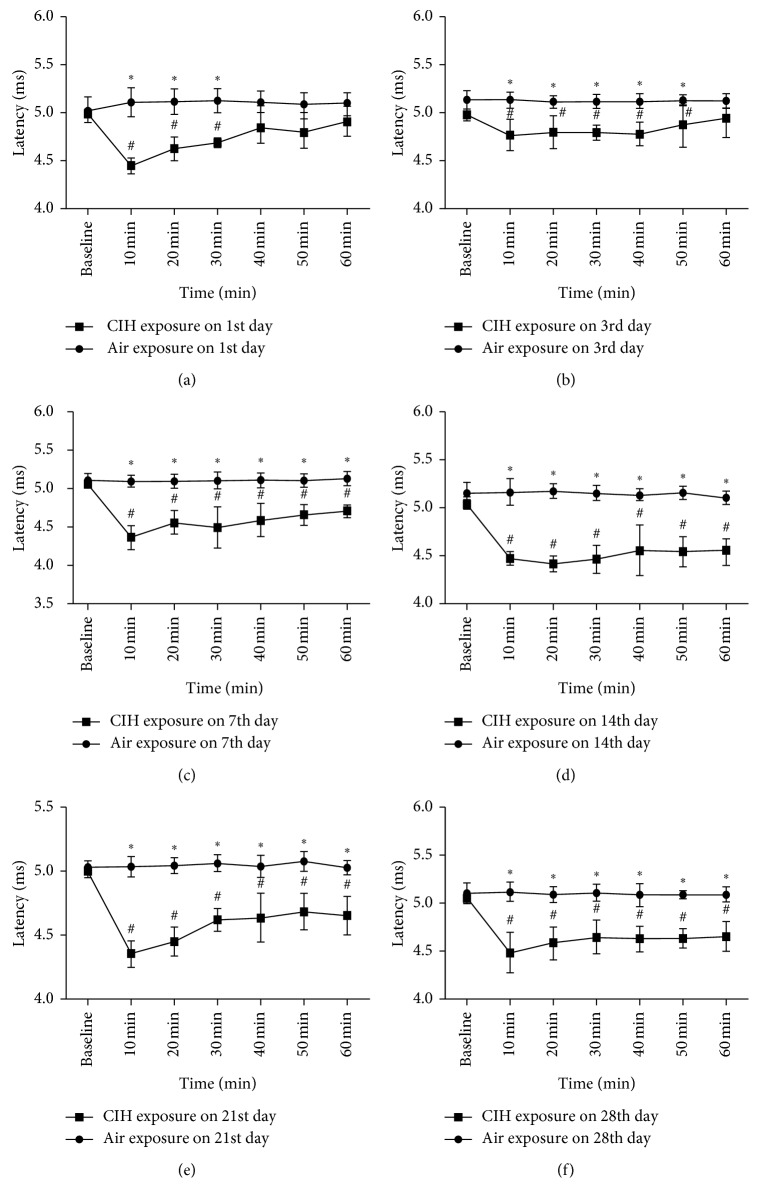
The comparison of TMS latency at different time points after IH stimulation among different groups. (a–f) Values obtained on the 1st, 3rd, 7th, 14th, 21st, and 28th days of the daily air/CIH exposure. ^∗^Difference between AIR and CIH groups at the same time point. ^#^Difference at different time points on each day in the CIH group.

**Figure 3:**
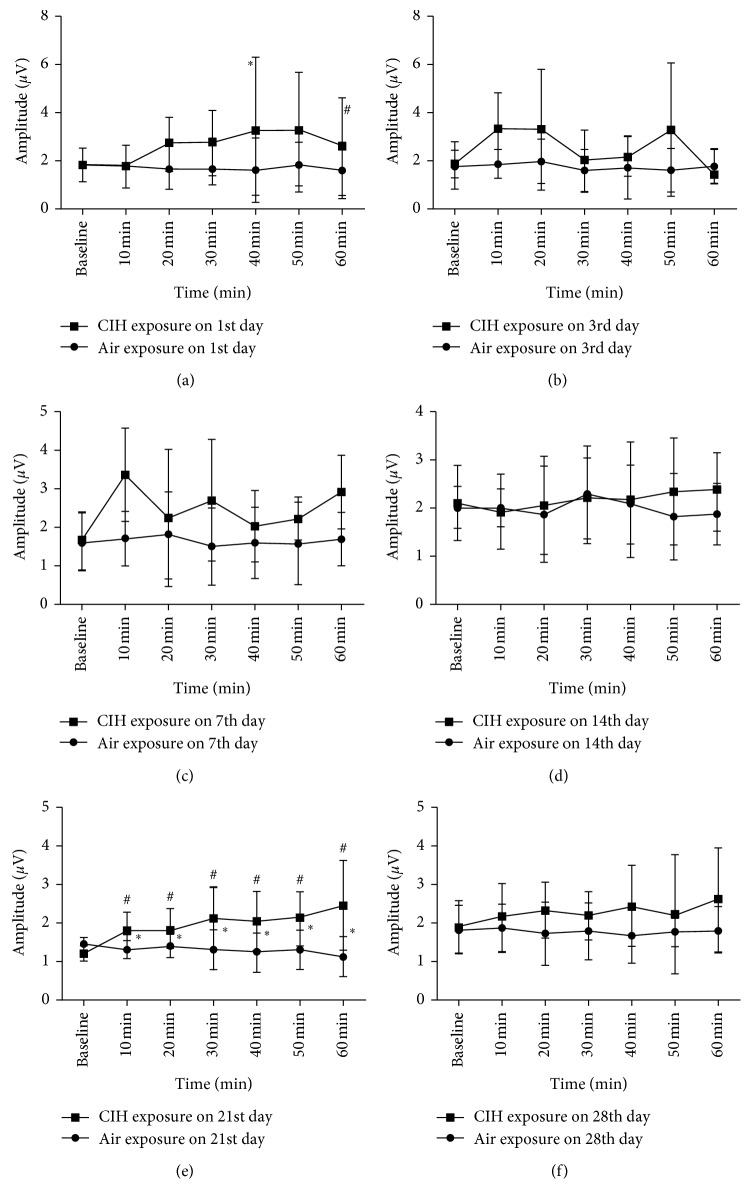
The comparison of TMS amplitude at different time points after IH stimulation among different groups. (a–f) Values obtained on the 1st, 3rd, 7th, 14th, 21st, and 28th days of the daily air/CIH exposure. ^∗^Difference between AIR and CIH groups at the same time point. ^#^Difference at different time points on each day in the CIH group.

The value of genioglossus latency detected by TMS was highly reproducible, with an average coefficient of variation of 5.5%. In terms of TMS latency recorded in the CIH group, it significantly decreased from 10 to 30 min after hypoxia stimulation on the 1st day of CIH, when compared with the baseline value (4.46 ± 0.01, 4.64 ± 0.15, 4.71 ± 0.05, and 5.03 ± 0.05 for 10, 20, 30 min, and baseline value, resp.; *p* < 0.05) and the air group (4.46 ± 0.01 versus 5.13 ± 0.09, 4.64 ± 0.15 versus 5.14 ± 0.11, and 4.71 ± 0.05 versus 5.14 ± 0.08 for 10, 20, and 30 min, resp.; *p* < 0.05). At the 7th day of CIH, TMS latency decreased from 10 min after hypoxia and this decrement lasted for the following 50 min. This persistence of the decrement of latency was observed for the following 3 weeks (Figure 2). Besides, TMS latency reached the valley point (4.37 ± 0.09) at 10 min after hypoxia on the 21st day of CIH. There was no statistical difference of the value detected at 60 min among each day for TMS latency. However, TMS amplitude only showed LTF at the 21st day of CIH (Figure 3).

In terms of TMS amplitude, the difference between the air and CIH groups was only observed from 10 min to 60 min after IH on the 21st day (60 min; *p*=0.023).

## 4. Discussion

This study was designed to explore the LTF of genioglossus EMG and its corticomotor activity at different stages of CIH in rats. We observed that a decreased TMS latency lasted for 60 minutes after the IH from the 7th day to the 28th day in the CIH group, when compared with the air group. Meanwhile, the persistent increase of the TMS amplitude and the genioglossus EMG activity from 10 to 60 min after the IH was only observed on the 21st and 28th days of CIH, respectively. These results indicated that CIH could induce the LTF of genioglossus corticomotor activity, which occurs prior to the facilitation of the excitability of genioglossus muscle activity in rats.

As a special respiratory muscle, the alteration of the activity of genioglossus during CIH was of great concern because it plays an important role in exploring the pathogenesis and treatment of OSAS. In 2004, McKay et al. put the newborn rats to episodic hypoxia (5% oxygen for 5 min and three cycles) and found that genioglossus EMG progressively increased and lasted for 60 min after CIH [4]. Tu et al. observed the LTF of the discharge of the hypoglossal nerve after 4 weeks of CIH in anesthetized and vagotomized rats [3, 10]. Based on these findings, the present study further explored the change of genioglossus at the central control level and confirmed that CIH could evoke the LTF of genioglossus corticomotor activity. This augmented corticomotor excitability presented as early as the first week of CIH and persisted throughout the following 3 weeks of CIH. On the other hand, the increment of genioglossus EMG activity has not been observed until the 4th week of CIH. These indicated that CIH could induce the enhanced corticomotor excitability of genioglossus at the early stage of CIH, and this central compensation occurs prior to the augmented genioglossus activity. This study was the first to continuously observe the LTF of genioglossus EMG activity and its associated central control during CIH in rats.

The fact that the recurrent obstructive sleep apneas occur only during sleep suggests that OSAS patients may have neuromuscular compensation of the upper airway during wakefulness [12]. Recently, two unexpected benefits of IH had been recognized as to improve respiratory and nonrespiratory somatic motor functions and to increase growth/trophic factor expression in the central nervous system. It was well known that CIH could induce the LTF of not only ventilation but also respiratory muscle activity [13, 14]. Hu et al. reported that 4 weeks of CIH could lead to a steady increase of genioglossus EMG activity [15]. Klawe et al. also found that snorers and OSA patients presented higher genioglossus activity with progressive hypoxia [13]. What is the underlying mechanism associated with this CIH-induced alteration of genioglossus activity? Besides, it remains unknown whether the excitatory inputs to genioglossus are derived from the central control. Moss et al. had reported that when exposed to recurrent episodic hypoxia, neonates expressed a tolerance to a subsequent hypoxic stimulus with relative hypoventilation; this change might relate to an adaptation of the respiratory center, and this adjustment made in response to repeated hypoxic episodes is assumed to be advantageous to the subject [14]. Therefore, we assumed that the CIH-induced central compensation might occur prior to the facilitation of the excitability of the genioglossus EMG activity. In the present study, the observation that the occurrence of LTF at different levels was presented in an order of priority has confirmed our hypothesis. Moreover, this study showed that the increment of genioglossus corticomotor activity occurred rapidly after the exposure to CIH, and this augmented excitability persisted throughout the early stage of CIH.

Although the mechanisms underlying OSA are complex, there is general agreement that a sleep-related decline in upper airway muscle activity contributes to airway narrowing and/or collapse [16]. Compared with normal subjects, OSAS patients showed increased GG EMG activity during wakefulness, while it decreased during sleep [12]. Many scholars tried to strengthen the mechanical performance of genioglossus, the contraction of which could counterbalance the anatomically unfavorable and collapsible upper airway in OSA patients. Mwenge et al. reported that the electrical stimulation, by implanted electrodes in the sublingual nerve, could decrease the apnea-hypopnea index (AHI) in OSAS patients [17]. Behan et al. also applied the approach of tongue exercise to increase the serotonin excitability inputs into the hypoglossal nerve, which innervate the contraction of genioglossus [18]. Furthermore, the LTF of neural drive to upper airway muscles during sleep could minimize or prevent apneic events [19]. Consistent with this suggestion, hypoxic episodes in sleeping OSA patients could induce an upregulation of the genioglossus corticomotor excitability, which could possibly facilitate the contraction of the genioglossus and the maintenance of UA patency. Nevertheless, whether this upregulation of the genioglossus corticomotor excitability transfers into the promotion of the pharyngeal airway stability during sleep deserves to be further investigated.

The limitation of this study was listed as follows: firstly, it is regretting that we did not observe the LTF of genioglossus and its central control during sleep. In our previous studies, the central compensation was observed only during wakefulness in both CIH-exposed rats and OSAS patients. Edge et al. reported that CIH caused the respiratory instability by blunting ventilatory LTF in sleeping rats [20]. Thus, it is important to further study the LTF of genioglossus and its central control during sleep. Secondly, the IH protocols described in the previous literature vary considerably in the severity and duration of hypoxic episodes, the interepisode intervals, and the cumulative exposure time. The modest low-dose IH protocol we used in this study, hypoxia (10% O_2_ in N_2_ for 45 s) and normoxia (21% O_2_ in N_2_ for 72 s) every 180 s for 8 h/d, mimics the OSA disease in a moderate severity. It deserves further investigation on the alteration of corticomotor excitability using different IH protocols.

In summary, this study observed the LTF of genioglossus EMG activity and its central control during CIH in awake rats. The genioglossus corticomotor excitability occurred in the first week of CIH and lasted for three weeks, while the genioglossus EMG activity did not change until the fourth week. Our findings confirmed that CIH affected the central control of genioglossus, with potential implications for OSAS treatment.

## Supplementary Materials

Supplementary Materials
*Supplementary Table 1*. The value of TMS latency at different time points after IH stimulation among different groups. *Supplementary Table 2*. The value of TMS amplitude at different time points after IH stimulation among different groups. *Supplementary Table 3*. The value of genioglossus EMG activity at different time points after IH stimulation among different groups. *Supplementary Figure 1*. The oxygen concentration of oxycycler. *Supplementary Figure 2*. TMS stimulation site.
