# Supplementary material for: Chronic Intermittent Hypoxia Induces the Long-Term Facilitation of Genioglossus Corticomotor Activity
Source: Can Respir J. 2018 Apr 23;2018:5941429. doi: 10.1155/2018/5941429 (PMC5937571; doi:10.1155/2018/5941429)
Supplement: Supplementary Materials — Supplementary Table 1. The value of TMS latency at different time points after IH stimulation among different groups. Supplementary Table 2. The value of TMS amplitude at different time points after IH stimulation among different groups. Supplementary Table 3. The value of genioglossus EMG activity at different time points after IH stimulation among different groups. Supplementary Figure 1. The oxygen concentration of oxycycler. Supplementary Figure 2. TMS stimulation site. [file 5941429.f1.doc]

Table 1 The value of TMS latency at different time point after IH stimulation among different groups.

| TMS-latency | 10min | | 20min | | 30min | | 40min | | 50min | | 60min | |
| --- | --- | --- | --- | --- | --- | --- | --- | --- | --- | --- | --- | --- |
|  | CIH | AIR | CIH | AIR | CIH | AIR | CIH | AIR | CIH | AIR | CIH | AIR |
| Day1 | 4.46±0.1*# | 5.1±0.18 | 4.6±0.15*# | 5.1±0.16 | 4.71±0.05*# | 5.13±0.14 | 4.9±0.2 | 5.15±0.13 | 4.82±0.19 | 5.1±0.14 | 4.94±0.15 | 5.1±0.13 |
| Day3 | 4.7±0.17*# | 5.1±0.09 | 4.8±0.17*# | 5.1±0.07 | 4.81±0.08*# | 5.14±0.07 | 4.79±0.12*# | 5.14±0.08 | 4.89±0.24*# | 5.1±0.05 | 4.96±0.2 | 5.1±0.08 |
| Day7 | 4.33±0.17*# | 5.1±0.08 | 4.47±0.16*# | 5.1±0.1 | 4.41±0.28*# | 5.12±0.12 | 4.51±0.23*# | 5.11±0.1 | 4.59±0.15*# | 5.1±0.09 | 4.64±0.09*# | 5.13±0.1 |
| Day14 | 4.47±0.07*# | 5.1±0.14 | 4.41±0.08*# | 5.1±0.07 | 4.46±0.15*# | 5.16±0.08 | 4.55±0.26*# | 5.14±0.06 | 4.53±0.16*# | 5.1±0.07 | 4.54±0.14*# | 5.1±0.07 |
| Day21 | 4.36±0.09*# | 5.0±0.07 | 4.46±0.1*# | 5.0±0.05 | 4.6±0.08*# | 5.05±0.05 | 4.63±0.17*# | 5.02±0.08 | 4.67±0.13*# | 5.0±0.07 | 4.73±0.14*# | 5.0±0.05 |
| Day28 | 4.48±0.24*# | 5.1±0.11 | 4.57±0.18*# | 5.1±0.09 | 4.67±0.2*# | 5.12±0.09 | 4.63±0.15*# | 5.1±0.13 | 4.66±0.11*# | 5.1±0.04 | 4.67±0.18*# | 5.1±0.09 |

TMS, Transcranial magnetic stimulation; CIH, chronic intermittent hypoxia.

*: indicated the difference between AIR and CIH groups at the same time point

#: indicated the difference among different time on each day in CIH group.

Table 2 The value of TMS amplitude at different time point after IH stimulation among different groups.

| TMS-amplitude | 10min | | 20min | | 30min | | 40min | | 50min | | 60min | |
| --- | --- | --- | --- | --- | --- | --- | --- | --- | --- | --- | --- | --- |
|  | CIH | AIR | CIH | AIR | CIH | AIR | CIH | AIR | CIH | AIR | CIH | AIR |
| Day1 | 1.82±0.85 | 1.84±0.75 | 2.58±0.99 | 1.69±0.78 | 2.62±1.2 | 1.72±0.78 | 3.05±2.86 | 1.66±1.12 | 3.07±2.25 | 1.83±0.77 | 2.44±1.88 | 1.62±0.94 |
| Day3 | 3.33±1.46 | 1.9±0.59 | 3.27±2.46 | 1.99±0.89 | 2.04±1.25 | .1.61±0.9 | 2.16±0.77 | 1,76±1.28 | 3.28±2.7 | 1.63±0.88 | 1.47±0.37 | 1．81±0.73 |
| Day7 | 3.27±1.16 | 1.71±0.68 | 2.21±1.69 | 1.78±1.07 | 2.65±1.5 | 1.51±0.96 | 2.02±0.88 | 1.61±0.88 | 2.2±0.55 | 1.6±1.02 | 2.85±0.92 | 1.68±0.65 |
| Day14 | 1.9±0.8 | 2.0±0.41 | 2.05±1.04 | 1．86±1.01 | 2.19±0.86 | 2.27±1.03 | 2.17±1.23 | 2.07±0.84 | 3.36±1.14 | 1．83±0.91 | 2.35±0.83 | 1.88±0.65 |
| Day21 | 1.93±0.57*# | 1.36±0.28 | 1.99±0.62*# | 1.49±0.38 | 2.29±0.96*# | 1.31±0.63 | 2.25±0.93*# | 1.28±0.63 | 2.3±0.85*# | 1.35±0.61 | 2.72±1.39*# | 1.15±0.62 |
| Day28 | 2.19±1.07 | 1.84±0.77 | 2.43±0.9 | 1.68±1.01 | 2.23±0.78 | 1.74±0.91 | 2.55±1.3 | 1.61±0.9 | 2.29±1.9 | 1.73±0.46 | 2.77±1.66 | 1.8±0.77 |

TMS, Transcranial magnetic stimulation; CIH, chronic intermittent hypoxia.

*: indicated the difference between AIR and CIH groups at the same time point

#: indicated the difference among different time on each day in CIH group.

Table 3 The value of genioglossus EMG activity at different time point after IH stimulation among different groups.

| EMG-amplitude | 10min | | 20min | | 30min | | 40min | | 50min | | 60min | |
| --- | --- | --- | --- | --- | --- | --- | --- | --- | --- | --- | --- | --- |
|  | CIH | AIR | CIH | AIR | CIH | AIR | CIH | AIR | CIH | AIR | CIH | AIR |
| Day1 | 67.4±2.5 | 71.25±2.42 | 72.13±4.51 | 70.67±2.31 | 72.1±3.77 | 70.1±0.9 | 68.35±6.44 | 71.63±2.56 | 69.1±7.5 | 70.46±3.76 | 70.78±5.83 | 71.1±4.26 |
| Day3 | 72.4±5.52 | 73.3±4.42 | 68.78±7.56 | 72.3±5.33 | 71.73±4.39 | .72.27±4.92 | 72.6±4.07 | 72.93±4.43 | 75.9±6.13 | 70.93±0.93 | 75.6±6.46 | 73.1±1.9 |
| Day7 | 69.7±3.94 | 71.9±4.89 | 71.23±5.03 | 72.3±4.04 | 73.4±1.9 | 72.46±1.97 | 72.8±1.25 | 70.26±1.62 | 70.82±1.62 | 70.23±0.98 | 70.3±1.75 | 70.33±3.76 |
| Day14 | 82.65±11.48*# | 75.78±2.35 | 84±8.29*# | 74.77±1.08 | 81.1±9.2 | 74.9±2.92 | 81.68±8.32 | 75.77±3.36 | 81.25±4.45 | 76.8±1。63 | 79.3±5.25 | 76.33±2.52 |
| Day21 | 96.3±7.77*# | 73.7±3.13 | 87.±6.65*# | 73.73±3.11 | 86.25±5.28*# | 74.83±3.01 | 88.55±3.66*# | 71.1±2 | 85.02±4.33*# | 71.4±2.51 | 80.38±1.55 | 72±3 |
| Day28 | 104.57±3.43*# | 73±2.29 | 101.47±3.64*# | 72.32±2.58 | 99.02±4.96*# | 72.33±4.04 | 99.33±4.39*# | 71.67±3.79 | 95.5±6*# | 70.93±5 | 97.96±3.17*# | 72±3.61 |

*： indicated the difference between AIR and CIH groups at the same time point

#： indicated the difference among different time on each day in CIH group. No statistical difference was observed at 60min among different days in CIH group.

Supplement figure 1. The oxygen concentration of oxycycler.


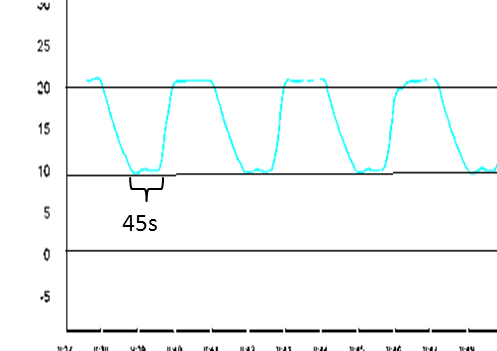


Hypoxia ( 10% O_2_ in N_2_ for 45 s ) and normoxia ( 21% O_2_ in N_2_ for 72 s ) every 180 s for 8 h/d ( from 8 a.m. to 4 p.m. ), Wall-like curve.

Supplement figure 2. TMS stimulation site.


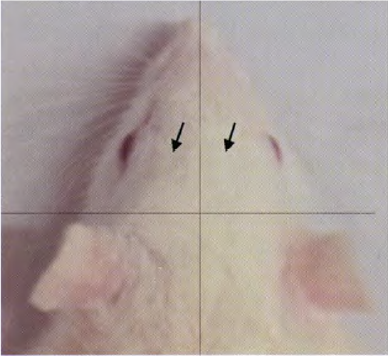


As shown by the arrow is the optimal coil position of TMS response.
